# Electrochemical Non-Enzymatic Detection of Glucose Based on 3D Electroformed Copper on Ni Foam Nanostructures

**DOI:** 10.3390/ma13122752

**Published:** 2020-06-17

**Authors:** Gheorghe Melinte, Andreea Cernat, Aurora Petica, Oana Lazar, Marius Enachescu, Liana Anicai, Cecilia Cristea

**Affiliations:** 1 Analytical Chemistry Department, Faculty of Pharmacy, Iuliu Haţieganu University of Medicine and Pharmacy, 4 Louis Pasteur St., 400349 Cluj-Napoca, Romania; Melinte.Gheorghe@umfcluj.ro (G.M.); Ilioaia.Andreea@umfcluj.ro (A.C.); 2 Center for Surface Science and Nanotechnology, University Polytechnica of Bucharest, Splaiul Independentei 313, 060042 Bucharest, Romania; aurora.petica@cssnt-upb.ro (A.P.); oana.lazar@cssnt-upb.ro (O.L.); marius.enachescu@cssnt-upb.ro (M.E.); lanicai@itcnet.ro (L.A.)

**Keywords:** glucose, modified electrodes, non-enzymatic detection, 3D electroformed Cu, electrochemical detection

## Abstract

Despite the fact that the electrochemical biosensors based on glucose oxidase represent the golden standard for the management of diabetes, the elaboration of nonenzymatic sensors became extensively studied as an out-of-the-box concept that aims to simplify the existing approach. An important point of view is represented by the low price of the sensing device that has positive effects for both end-users and healthcare systems. The enzyme-free sensors based on low-cost materials such as transition metals have similar analytical properties to the commercial ones while eliminating the issues associated with the presence of the enzyme, such as the stability issues and limited shelf-life. The development of nanoporous nanomaterials for biomedical applications and electrocatalysis was referred to as an alternative to the conventional methods due to their enlarged area, electrical properties, ease of functionalization and not least to their low cost. Herein, we report the development of an electrochemical nonenzymatic sensor for glucose based on 3D copper nanostructures with Ni foams as promotor of the enhanced nanoporous morphology. The sensors were successfully tested in the presence of the designated target, even in the presence of common interference agents found in biological samples.

## 1. Introduction

The World Health Organization recently reported more than 422 million people suffering from diabetes, especially in developing and poor countries [1]. Diabetes is a serious chronic disease that occurs in two cases: the pancreas is not able to produce enough insulin or the body cannot effectively use the insulin it produces [1]. 

Diabetes of all types is a serious health issue leading to complications and with an increased overall risk of dying prematurely. The most reported complications include heart attack, stroke, kidney failure, leg amputation, vision loss and nerve damage. Poorly controlled diabetes also increases the risk of fetal death and other complications during pregnancy. A successful approach of living well with diabetes is an early diagnosis, meaning that the longer a person lives with undiagnosed and untreated diabetes, the worse their health outcomes are likely to be. A very useful and affordable method that should be available in primary healthcare settings is the access to basic diagnostics, such as blood glucose (denoted Glu) testing. In this case, validated systems for referral and back-referral are needed, as patients will need periodic specialist assessment or treatment for complications. For patients with diabetes, several cost-effective interventions can improve their outcomes, irrespective of what type of diabetes they may have. These interventions include Glu exact control, through a combination of factors like diet, physical activity and, if necessary, medication; control of blood pressure and the amount of lipids in order to reduce the cardiovascular risk and other complications; and regular screening for damage to the eyes, kidneys and feet aiming to facilitate early treatment. For diabetes management the use of standards and protocols is highly recommended and most important [1,2]. Therefore, a continuous monitoring of the body Glu level is required in order to reduce the mortality rate and to maintain the physical body characteristics. Blood sugar monitoring devices are commonly and currently used to properly manage diabetes.

A large range of techniques are involved to measure the Glu concentration, including optical [3], coulometric [4], capacitive detection [5], spectrophotometry, high-performance liquid chromatography, Raman, infrared spectroscopy (IR), electronic, fluorescent, acoustic and transdermal ones [6,7,8].

Currently, most Glu biosensors are of the electrochemical type due to their enhanced sensitivity, reproducibility, simplicity of use, easy maintenance and lower cost [2,6,9]. Most of the commercially available Glu biosensors are based on the use of enzyme-based electrochemical detection involving amperometric methodology, and they have been widely investigated over the last few decades [6,9]. All types of enzyme-based biosensors require laborious and complex enzyme immobilization procedures which induce an uncertainty related to the artificially engineered biological substances, regardless of the type of the enzymes. Therefore, in spite of their high Glu selectivity, they suffer from reproducibility, instability, deactivation due to long time storage and exposure to elevated temperatures, as well as from a relatively higher cost [9,10,11]. Under these circumstances, the development of non-enzymatic electrochemical sensors without biological functional units has attracted an increased interest thanks to their structural simplicity, lower cost and better quality control for mass production, while benefitting from the current development of a large range of novel nanostructured materials possessing various morphologies [9,11,12,13,14] 

The rapid advancement in nanoscience and nanotechnology has opened new approaches for the preparation of nanostructured electrodes either as films or as nanoparticles, nanowires, nanorods or nanotubes, which are usually characterized by electrocatalytic properties due to the enlargement of surface area, generation of electrocatalytic active sites and formation of nano-space enclosed by conducting surfaces [2,9,11,15,16,17,18,19,20,21].

Single and binary noble metal nanomaterials as well as their hybrid with carbon-based nanomaterials have been widely studied to build electrodes for non-enzymatic Glu detection, including Pt [22,23,24,25] Au [26,27,28], Pt-Au nanocorals [29], Pt-Pd nanoflakes [30] and nanoporous PtAg [31]. Although noble metals show high sensitivity towards Glu oxidation, the surface poisoning due to the adsorption of intermediates and chloride ions remains a challenge. In addition, a major drawback is their high cost, which makes them less competitive in practical applications.

There has been a growing interest in the fabrication of non-enzymatic glucose Glu sensors based on cheaper non-precious transition metals [9,32]. Particularly, Ni-, Cu- and Co-based metal and metal oxide structures have been studied due to their good catalytic performance [11,19,21,32,33]. Various Cu, Cu compounds and Cu alloy nanomaterials possessing different morphologies have been investigated as electrodes for Glu electrochemical detection, including Cu nanowires [2,9,16,17], Cu(OH)_2_ nano-flowers [34] or nano-tubes [35], Cu_2_O or CuO-based nanostructures [36,37], as well as Cu/Co bilayers [19] and Cu/Ni nanostructures [38,39]. Compared to other types of morphologies, i.e., nanowires, nanorods, nanotubes, etc., the development of novel nanoporous materials have also attracted an increased interest for applications in electrocatalysis and electroanalysis. Besides an enlarged specific surface area, they possess other interesting properties such as the nano-confinement effect, electrical layer overlapping discriminative electrokinetics, ion-selective impedance, etc. [40,41]. In addition, functionalization of nanoporous materials by applying additional coatings of metals or oxides may also enhance the electrocatalytic or electroanalytical effect [42,43].

Considering everything discussed above, this paper aims to assess a novel sensing platform for the non-enzymatic electrochemical detection of Glu based on 3D electroformed copper nanostructures with enhanced porosity. Therefore, the electrochemical preparation and characterization of 3D Cu nanoporous electrodes were initially presented. The prepared electrodes were then investigated as the main component of the Glu sensor involving chronoamperometry. Their linear range, detection limit and selectivity are determined and discussed.

## 2. Materials and Methods 

### 2.1. Reagents and Instruments

Nickel chloride (NiCl_2_∙6H_2_O), copper sulfate (CuSO_4_∙5H_2_O), ammonium chloride (NH_4_Cl), sulfuric acid (H_2_SO_4_), nitric acid (HNO_3_), potassium ferro/ferricyanide ([Fe(CN)_6_]^3‒/4‒^), sodium hydroxide (NaOH), D-(+)-Glu (C_6_H_12_O_6_), paracetamol (P), ascorbic acid (AA), Rhamnose (R) and uric acid (UA) were purchased from Sigma-Aldrich, Darmstad, Germany and used as received. The commercial human serum was purchased also from Sigma-Aldrich and kept at ‒20 °C. All reagents were at least of p.a. grade and the solutions were prepared using Milli-Q ultrapure water (18 MΩ/cm).

Electrochemical investigations were performed involving PARSTAT 4000 (Princeton Applied Research, AMETEK Scientific Instruments, Oak Ridge, TN, USA) ), Autolab PGSTAT302N (Metrohm, Eco Chemie, The Netherlands) and Palmsens EmStat Blue potentiostats PalmSens BV, GA Houten, The Netherlands), using a three-electrode cell where a platinum wire (BASi, West Lafayette, IN, USA) and a Ag/AgCl electrode (BASi, West Lafayette, IN, USA) served as the counter and reference electrode, respectively. Cu strip and different Cu nanoporous morphologies acted as working electrodes. The working volume was 5 or 10 mL solution under continuous stirring using a magnetic stirrer FALC F70, 100–1800rpm (Falc Instruments, Treviglio, Italy).

The morphology and elemental composition of the prepared 3D electroformed copper nanostructures were examined using scanning electron microscopy (SEM), associated with energy dispersive X-ray spectroscopy (EDX) (SU8230, HITACHI High-Technologies Corp., Tokyo, Japan equipped with EDX Oxford detector analyzer, Oxford Instruments NanoAnalysis & Asylum Research, High Wycombe, UK). The chemical composition and crystal phase of the porous nanostructures were determined using X-ray diffractometry (XRD) (High Resolution SmartLab X-ray diffractometer Rigaku, Tokyo, Japan, 9 kW, with rotating anode,) using CuK radiation, at a speed of 2 s/step (1step = 0.05°). 

### 2.2. Preparation of the 3D Cu Nanoporous Electrodes

The Cu nanoporous electrodes were electrochemically synthesized onto Cu metallic strips, initially subjected to surface preparation consisting of mechanical polishing using 1000 and 2000 abrasive paper, followed by chemical pickling in 1:1 HNO_3_:H_2_O solution at 25 °C for 20–30 s, then rinsed with running water and distilled water and air dried.

The selected electrodeposition route to obtain the Cu nanoporous electrodes consisted of (i) the electrochemical growth of Ni foams on Cu involving the dynamic hydrogen bubble template according to [44,45], followed by (ii) Cu deposition using a classical acidic sulfate type electrolyte. The compositions of the used electrolytes and the operation conditions are summarized in Table 1.

### 2.3. Electrochemical Assessment of 3D Cu Nanoporous Electrodes

Preliminary experimental investigations involving electrochemical impedance spectroscopy (EIS) in 5 mM [Fe(CN)_6_]^3−/4‒^ dissolved in 0.1 M NaOH solution, as redox probe, on a frequency window of 10 mHz to 100 KHz at open circuit potential were performed on Cu strip and Cu-based nanoporous coating systems having a constant geometrical area of 0.63 cm^2^, in order to get more information on their electrochemical characteristics. The fitting of the impedance data was performed using ZView 2.4 software from Scribner Association Inc., Derek Johnson (Southern Pines, NC, USA). In addition, cyclic voltammetry (CV) investigations were also done in the same solution, for scan rates between 10 and 50 mV/s.

CV experiments in 0.1M NaOH solution in the absence and in the presence of 0.225 mM Glu were recorded to assess the electrochemical response for this analyte of the investigated Cu strip and Cu-based nanoporous electrodes. The potential was swept twice from 0 V to 0.8 V with a scan rate of 10 mV/s.

The detection of Glu was performed using chronoamperometry (CA) at +0.55 V vs. Ag/AgCl in 5 mL 0.1 M NaOH solution under continuous stirring at 500 rpm (interval time 0.1 s, analysis time: up to 1200–1600 s), with successive additions every 100 s of 2.5 mM analyte solution (the first 4 additions of 12.5 µL, followed by 50 µL of the standard solution) or 25 mM Glu (50 µL additions). 

The interference experiments were performed using the same protocol in the presence of several electroactive species, respectively: paracetamol (P), ascorbic acid (AA), Rhamnose (R) and uric acid (UA). The additions of the interfering species (a final concentration of 20 µM) were alternated with those of the analyte (a final concentration of 400 µM) to confirm the selectivity of the platform. The experiments were performed on a working surface area of 25 mm^2^.

The studies on real samples were performed on commercial human serum after a dilution step 1:100 with 0.1 M NaOH. CA at +0.55 V vs. Ag/AgCl was performed in 10 mL NaOH with successive additions every 50 s of 0.1 M Glu in different volumes of 0.1 M NaOH (10 µL, 20 µL, 40 µL, 60 µL, 80 µL, each volume 4 times). The experiments were performed on a working surface area of 6 mm^2^.

All electrochemical experiments were conducted at room temperature.

## 3. Results and Discussions

### 3.1. Preparation and Characterization of 3D Cu Nanoporous Electrodes

**Figure 1:**
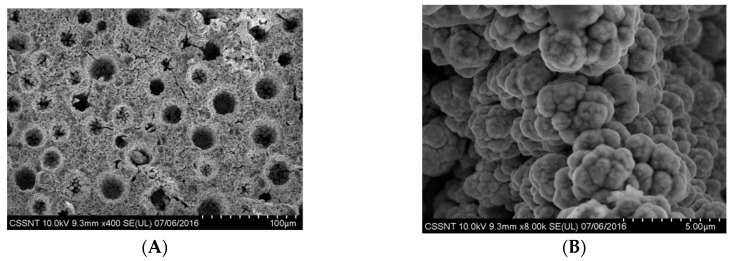
SEM micrographs at different magnifications for electrodeposited Ni_f_ (1 A/cm^2^, 3 min.) onto Cu metallic substrate. (**A**) × 400, (**B**) × 8000.

Electrodeposition of Ni foams on Cu substrates involving the chloride type electrolyte having the composition shown in Table 1 facilitated the formation of porous structures with different sizes of the pore’s diameter, depending on the applied current density and deposition time. Uniform, dark grey layers were formed, regardless of the applied preparation parameters. A typical morphology of the electrodeposited Ni foam is illustrated in Figure 1.

The development of this type of structure is mainly determined by the hydrogen bubbling occurring simultaneously to the electrochemical reduction of nickel ions, acting as a dynamic template. This type of morphology possesses a large specific surface area facilitating an enhancement of the electrocatalytic or electroanalytical response [37,40,43,44,45]. As shown in Figure 1, a relatively uniform porous structure may be noticed, presenting both macroporosity (pore diameters of about 25–35 μm) and microporosity (pore diameters of about 3–5 μm) features. In addition, the deposits show a nodular growth, leading to a cauliflower-like morphology.

In order to get more information on the distribution of the Ni foam pore diameter sizes, a statistical analysis was performed and the pore diameters were extracted from a low magnification SEM image by measuring around 150 individual pores. The histogram was best fitted with a Gauss function (not shown here), exhibiting a unimodal distribution of pore diameters. The highest number of pores were found to be within a 20 and 45 μm diameter size range. The mean diameter size of the pores was calculated as 31.25 ± 8.4 μm.

It is well known that the electrochemical deposition is an atomic/molecular-level process so that a deposited layer entirely takes the three-dimensional shape of the substrate with a very high accuracy. Therefore, electrodeposition onto a 3D architecture will keep the substrate profile [44,45,46]. Moreover, the subsequent electrodeposition of Cu from the acidic electrolyte combines the strong porous support with the high conductivity and reactivity of Cu.

**Figure 2:**
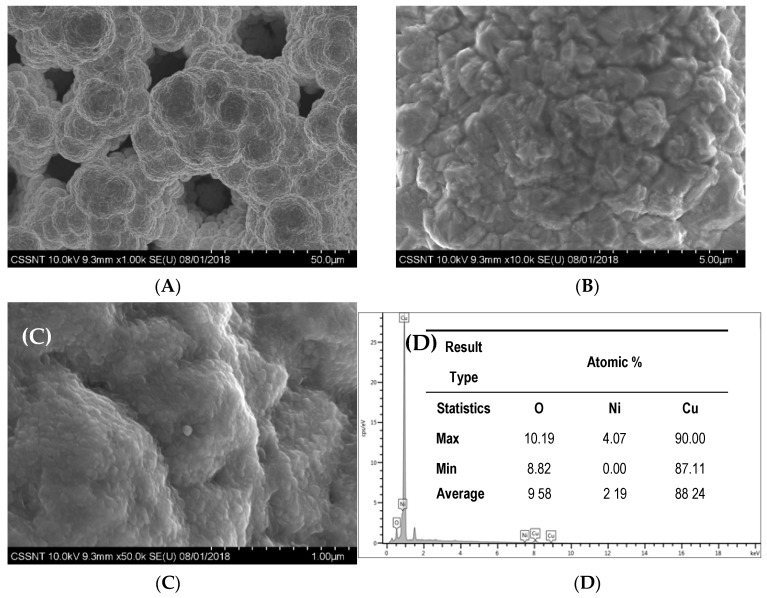
SEM micrographs at different magnifications (**A**: × 1000; **B**: × 10,000; **C**: × 50,000) and corresponding EDX analysis (**D**) of Cu electrodeposited onto Ni foam at 35 mA/cm^2^ for 15 min.

Figure 2 presents an example of the obtained morphology of Cu electrodeposited onto Ni foam substrate and the corresponding EDX spectrum. As expected, the porous structure of genuine Ni foam is clearly evidenced. Pores having diameters in the range of 10 to 20 μm and an internal structure of the Cu deposit possessing a globular-like structure with an average diameter of about 100 nm may be noticed.

**Figure 3:**
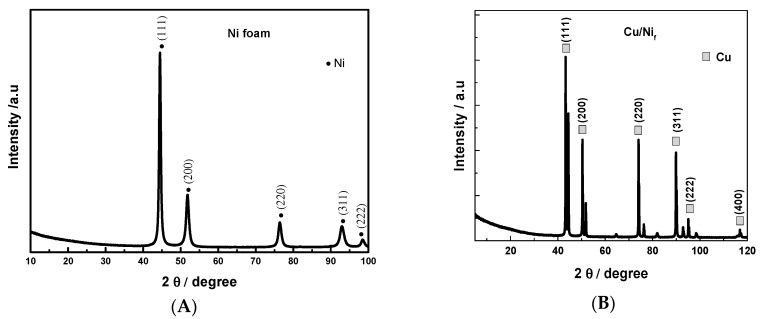
X-ray diffractograms for electrodeposited Ni foam at 1 A/cm^2^ for 3 min. (**A**) and for Cu/Ni_f_ electrode (**B**) (Cu electrodeposited onto Ni foam at 35 mA/cm^2^ for 15 min.).

The EDX analysis of the Cu/N_f_ electrode in Figure 2 revealed the presence of Cu and Ni elements besides O, where the atomic content of Ni was far less than that of Cu, suggesting the Cu deposit successfully covered the Ni_f_ substrate. The chemical composition and crystal phase of the electrodes was examined using XRD analysis, as shown in Figure 3. 

The electroformed Ni foam showed Ni characteristic peaks at 44°, 51°, 76°, 92° and 98°, corresponding to the (111), (200), (220), (311), and (222) reflections of the f.c.c. structure [47]. After Cu electrodeposition onto Ni_f_, new diffraction peaks appearing in the XRD pattern can be well indexed to the (111), (200) and (220) crystal planes of the f.c.c. polycrystalline copper standard (PDF card No. 00-004-0836).

The resulting Cu/Ni_f_ electrode was then subjected to electrochemical characterization.

### 3.2. Electrochemical Characterization of 3D Cu Nanoporous Electrodes

**Figure 4:**
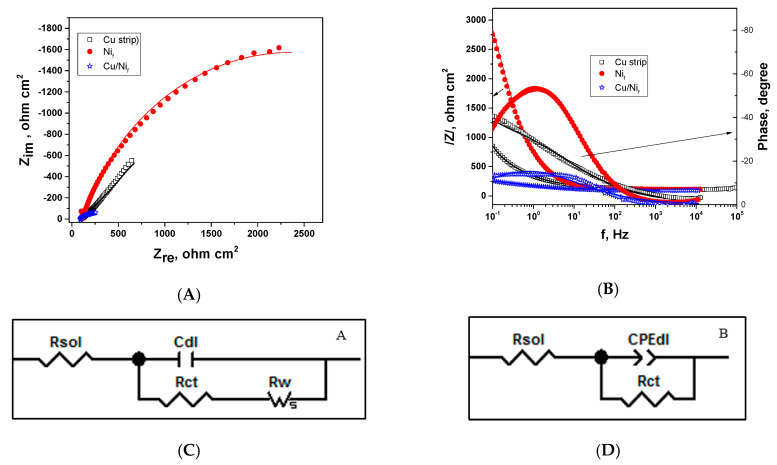
EIS data recorded in 5 mM [Fe(CN)_6_]^3‒/4‒^ in 0.1 M NaOH solution for Cu strip, Ni_f_ and Cu/Ni_f_ electrodes as Nyquist (**A**) and Bode (**B**) plots. A and B represent the proposed equivalent circuit to fit the measured points. (**C** and **D**)

The interfacial charge-transfer phenomena on the electrodes was investigated involving EIS measurements using an electrolyte consisting of 5 mM [Fe(CN)_6_]^3−/4−^ in 0.1 M NaOH, at open circuit potential. Figure 4 comparatively presents the recorded EIS spectra for Cu strip, Ni_f_ and Cu/Ni_f_ electrodes as Nyquist and Bode plots.

A single very small semicircle followed by a straight line at low frequency is evidenced in the Nyquist plot of the Cu strip electrode, usually characteristic of diffusive processes, showing the very low charge transfer resistance (*R_ct_*) of the Fe(CN)_6_^3-/4-^ in slightly alkaline medium in agreement with [48].

The Ni foam electrode exhibits a broadened semicircle, assigned to the porous surface presence. As expected, the Cu/Ni_f_ electrode presents a very low semicircle, suggesting an improvement of the electron transfer rate.

In order to fit the experimental data, simple equivalent circuit models have been used (as shown in Figure 4). In the case of the Cu strip and Cu/Nif electrodes, the equivalent circuit model A was involved, comprising the solution resistance (*R_sol_*), in series with a combination between the double-layer capacitance (*CPE_dl_*) in parallel with the charge transfer resistance (*R_ct_*). To take into account the diffusion process that occurs through the metallic layer, a Warburg element (R*_W_*) was added. The fitting of experimental data related to the Ni_f_ electrode was performed involving the equivalent circuit model B, consisting of the solution resistance (*R_sol_*), in series with a combination between a constant phase element corresponding to the double-layer capacitance (*CPE_dl_*) in parallel with the charge transfer resistance (*R_ct_*). During fitting, double-layer capacitances were replaced by a constant phase element (*CPE*). In general, the use of *CPE* is appropriate when inhomogeneities are present at atomic or molecular level, including the surface roughness/porosity, diffusion or adsorption [49]. The coefficient T (F s^n−1^/cm^2^) was used as capacitive parameter. The selected equivalent circuit showed quite good agreement between the experimental data (symbols) and simulated data (solid lines).

The values of impedance parameters, which are obtained by fitting the impedance data with ZView software using the equivalent circuits proposed in Figure 4, are listed in Table 2.

As can be seen from Figure 4, the value of *R_ct_* corresponding to the Ni_f_ electrode as substrate significantly decreased as the Cu layer was deposited, from around 4500 Ω towards 10 Ω, suggesting an improvement of the electron transfer rate. Therefore, the presence of the supplementary metallic Cu on the foam layer improved the electron transfer rate, highlighting the properties of the new electroformed porous material. In addition, the overall resistance including R_W_ in the case of the Cu strip exceeds the value obtained in the case of the Cu/Ni_f_ electrode, showing an improved electron transfer rate, too.

From the calculated double-layer capacitances, *C_dl_*, and taking into consideration the average double-layer capacitance of a smooth metal surface of 20 µF/cm^2^ [50], the real surface area (*A_real_*) may be calculated as

(1) $$A_{real}=\frac{C_{dl}}{20}\;[{\mathrm{cm}}^{2}]$$

The roughness factor, σ, which is related to the real to geometrical surface area (*A_geometric_*) ratio, is calculated as

(2) $$\sigma \;=\;\frac{A_{real}}{A_{geometric}}$$

Table 3 presents the double-layer capacitance, real surface area and corresponding surface roughness values for the investigated electrodes calculated from the EIS data.

As expected, the use of the Ni foam porous substrate facilitated higher roughness values, which may further exhibit a positive influence on the electroanalytical characteristics of the electrode, in agreement also with data reported in [45]. In addition, an increase of the roughness factor was noticed in the case of the Cu/Nif electrode, which may be attributed to the Cu electrodeposition procedure which involved an additive-free electrolyte, thus leading to a slightly more porous surface, in agreement with [51].

**Figure 5:**
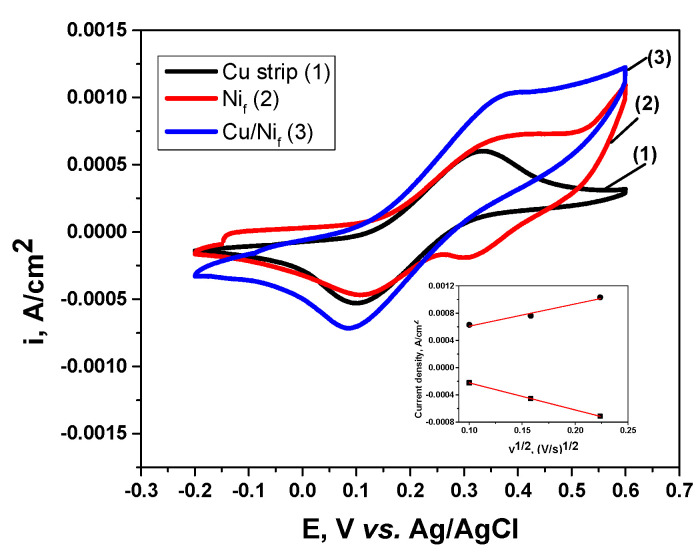
CVs recorded in 5 mM [Fe(CN)_6_]^3‒/4‒^ in 0.1 M NaOH solution for Cu strip, Ni_f_ and Cu/Ni_f_ electrodes (scan rate = 50 mV s^‒1^). The inset shows the dependence of the anodic and cathodic peak currents vs. the square root of the scan rate for the Cu/Ni_f_ electrode.

In order to get more information on the electrochemical characteristics of the Cu/Nif proposed electrode, comparative cyclic voltammetry investigations were performed at various scan rates between 10 and 50 mV/s involving 5 mM [Fe(CN)_6_]^3‒/4‒^ in 0.1 M NaOH solution, as exemplified in Figure 5. Usually, the ferrocyanide/ferricyanide redox couple is used as the model redox couple. This is considered a quasi-reversible couple [52] and is very stable during electrochemical experiments.

As observed, for all three electrodes (Cu strip, Nif and Cu/Nif), the potential of the anodic and cathodic peaks is approximately the same, whatever the chemical nature of the electrode surface. Furthermore, the peak currents for the three electrode materials are quite similar for both electrode processes (anodic and cathodic). However, as expected the corresponding currents of the redox peaks are the highest for the Cu/Nif electrode, suggesting an improvement of the redox activity.

The recorded CV in the case of the Nif electrode shows the presence of two peaks on the cathodic branch. The first peak, located at around 0.3–0.35 V (vs. Ag/AgCl), presented a current plateau at low scan rates (10 mV/s, not shown here) and becomes more evident as the scan rates increased. According to the literature [48,53], [Fe(CN)_6_]^3‒^ serves as an electron acceptor favoring the oxidation of a Ni foam surface to β-Ni(OH)_2_ and further undergoes electrooxidation at high positive potentials to form NiOOH with no anodic peak appearance. During the reverse scan, the [Fe(CN)_6_]^4‒^ acts as an electrons donor providing the required electrons for reduction of NiOOH, thus activating the redox reactions of the Nif electrode. Therefore, the cathodic peak on the recorded CV for the Nif electrode (curve 2 from Figure 5) might be assigned to NiOOH reduction in alkaline medium with Ni(OH)_2_ re-formation.

The second cathodic peak on the recorded CV for the Nif electrode at around 0.15 V (vs. Ag/AgCl) may be attributed to the cathodic process of the ferrocyanide/ferricyanide couple, in agreement also with [48].

The anodic peak to cathodic peak potential separation, ΔEp, was about 150–200 mV for the investigated electrodes, suggesting a quasi-reversible process [52]. The values of ΔEp higher than 59 mV (for one transferred electron) might be attributed to the electrolysis conditions (the solution conductivity induces an uncompensated IR voltage drop, which increases as the scan rate is higher).

The inset of Figure 5 illustrates an example of the dependence of the anodic and cathodic peak currents against the square root of the scan rate for the Cu/Nif electrode. The linear relation proves that this is a diffusion-controlled process for both anodic and cathodic scans. It is worth mentioning that additional experiments presented the same linear dependence was noticed for Cu strip and Nif electrodes, too (results not shown).

**Figure 6:**
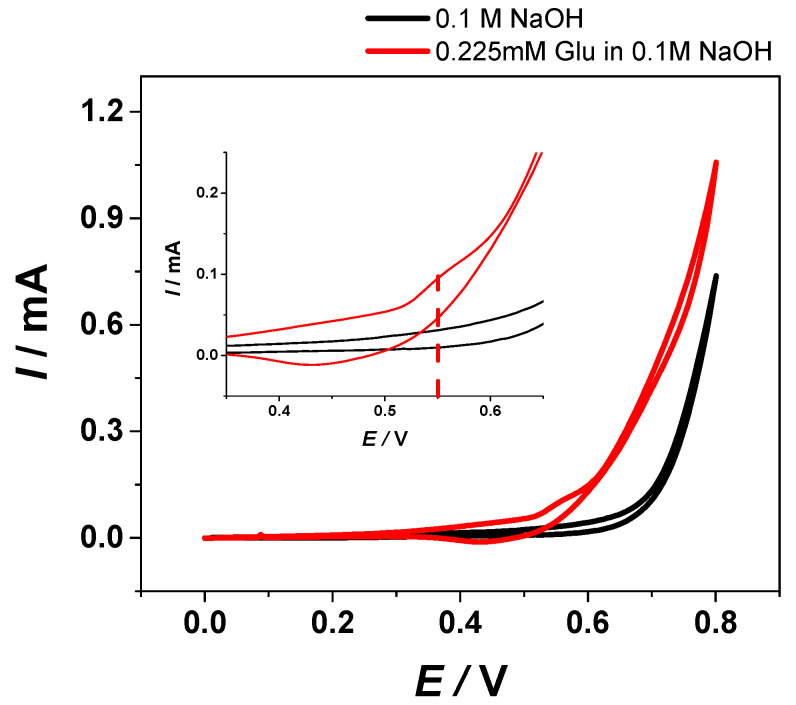
CV of the Cu/Ni_f_ working electrode in the absence (black line) and in the presence of 0.225 mM Glu (red line) in 0.1 M NaOH aqueous solution (scan rate: 10 mV/s, working electrode surface area of 25 mm^2^).

The properties of the Cu/Ni_f_ electrodes were evaluated in the presence of Glu, as the model molecule. Figure 6 presents the recorded CV in 0.1 M NaOH electrolyte in the absence and in the presence of 0.225 mM Glu at a scan rate of 10 mV/s. 

As can be seen from Figure 5, during the anodic scan, an increase of the current was observed at +0.55 V vs. Ag/AgCl in the presence of 0.225 mM Glu, assigned to the Glu oxidation. In addition, the slight increase in the capacitive current corresponds to the electrochemical oxidation of the target analyte. When the same experiment was performed in the absence of Glu, no peak was noticed.

Therefore, the potential value of +0.55 V vs. Ag/AgCl was employed for the evaluation of the analytical performance of the Cu/Ni_f_ electrode for the electrochemical detection of Glu using steady state amperometric measurements in 0.1 M NaOH solution.

**Figure 7:**
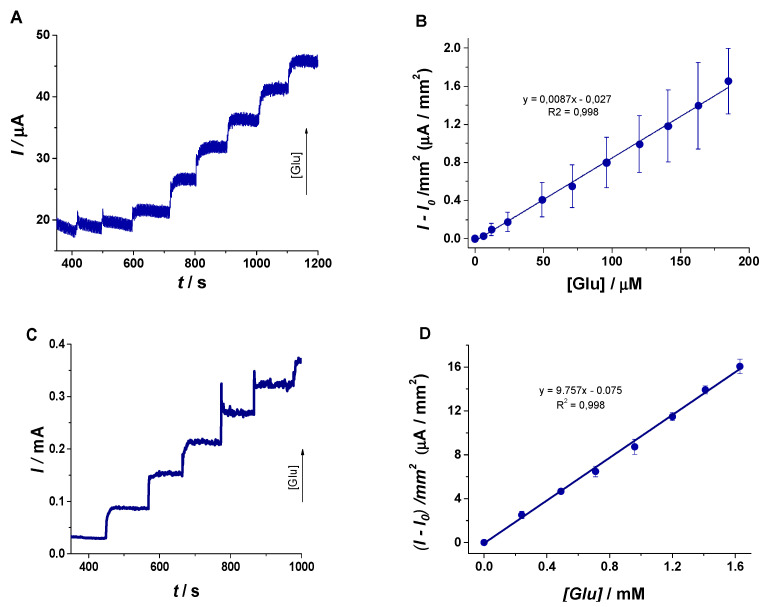
(**A**) CA of CuNi_f_ electrodes in 0.1 M NaOH with standard additions of 2.5 mM Glu every 100 s (4 times of 12.5 µL to reach small concentration difference and then 25–50 µL until the end of analysis), under continuous stirring at 500 rpm (0.55 V, 0.1 s step potential, 1200 s analysis time); (**B**) the calibration curve in the range of 6–185 µM Glu; (**C**) CA of CuNi_f_ electrodes in 0.1 M NaOH with standard additions of 25 mM Glu every 100 s of solution (50 µL) under continuous stirring at 500 rpm (0.55 V, 0.1 s step potential); (**D**) the calibration curve in the range of 0.24–1.63 mM Glu.

As presented in Figure 7, the signal was stabilized for 400 s followed by standard additions of 2.5 mM (12.5–25 µL) and 25 mM Glu (50 µL) solutions in 0.1M NaOH every 100 s. The signal corresponding to the electrochemical oxidation of Glu followed a stair-like augmentation trend. It is clearly visible that the signal stabilized in almost 5 s after the addition of the standard solution, suggesting that the presence of the porous morphology of the Cu/Ni_f_ electrode improved the electrochemical transfer rate and allowed the rapid oxidation of the analyte. 

When plotting I-I_0_/mm^2^ against Glu concentration in the case of the experiments performed on standard solutions, a proportional increase was observed (see Figure 7A,C). Two calibration curves were obtained for the elaborated platform, as follows:

(i) The first one in the range of 6 – 206 µM defined by the equation y(µA/mm^2^) = 0.0087 × [Glu(µM)] ‒ 0.027 (R^2^ = 0.998) has the role to demonstrate the analytical performances of the sensor, with a limit of detection (LOD) calculated as signal/noise = 3 of 2 µM and a limit of quantification (LOQ) of 6 µM.
(ii) The second one in the range of 0.24–1.63 mM defined by y(µA/mm^2^) = 9.757 × [Glu(mM)] ‒ 0.075 (R^2^ = 0.996) underlines the possibility to employ the sensor in real scenarios. The normal level of blood Glu is found to be in the 3–5.5 mM range, and in this case, the sensor could be successfully used to assess even hyperglycemic pathological levels after the dilution of the sample with an electrolyte solution (1:10 or even 1:100). Thus, the calculated concentrations will be found in the linear range and, taking into account the dilutions step, the real value of blood Glu could be determined. Further studies will be performed in order to extend the analytical performances.

**Figure 8:**
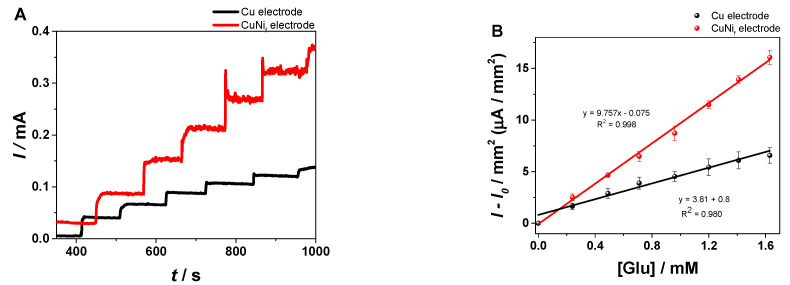
(**A**) CA on Cu strip electrodes (black line) and Cu/Ni_f_ on Cu substrate electrodes (red line) in 5 mL 0.1 M NaOH with standard additions of 25 mM Glu every 100 s (50 µL under continuous stirring at 500 rpm) (0.55 V, 0.1 step potential, 1200 s analysis time, 500 rpm). (**B**) The corresponding calibration curves for the Cu bare electrode (black line) and Cu/Ni_f_ on Cu electrode (red line).

A control experiment was performed on the bare Cu strip electrode having a similar geometrical surface, in order to evaluate the impact of the presence of the porous morphology due to the Cu/Ni_f_ substrate on the detection of Glu. The experiments were performed in the presence of 25 mM Glu in order to be compared with the data obtained on the Cu/Ni_f_ electrode for the second linear range close to the physiological levels of Glu. According to the CA and calibration curves presented in Figure 8, it is clearly observed that the sensor based on metallic foam has a two-fold higher sensitivity than in the case of the unmodified one. Thus, the Cu/Ni_f_ configuration yielded more reliable results for Glu detection.

### 3.3. Interferences Studies

Selectivity is one of the most important parameters that characterizes an amperometric sensor and it shows the sensors capacity to detect the target analyte from a complex matrix. Interferences studies were carried out in the presence of common analytes found with Glu in biological fluids, such as UA, P, AA and sugars like R. 

**Figure 9:**
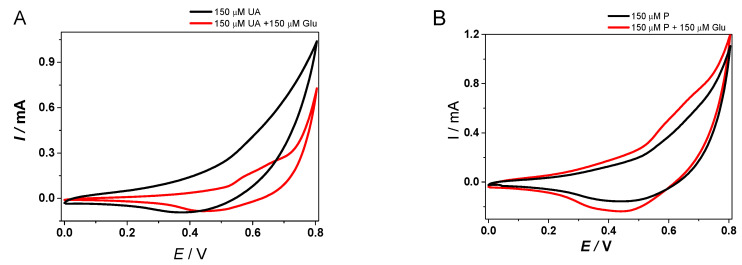
CV in 0.1 M NaOH solution in the presence of 150 µM UA (**A**) and 150 µM P (**B**) as interfering agents in the absence (black line)/presence (red line) of 150 µM Glu. (0–0.8 V, scan rate 25mV/s, working electrode surface area of 25 mm^2^).

The selectivity of the Cu/Ni_f_ platform for Glu was firstly assessed using CV recorded in 0.1 M NaOH solution in the presence of 150 µM interfering molecule, in the absence/presence of 150 µM Glu (Figure 9). 

It can be clearly seen that on the Cu/Ni_f_ configuration the presence of both UA and P did not interfere with the anodic signal corresponding to the electrochemical oxidation of Glu.

**Figure 10:**
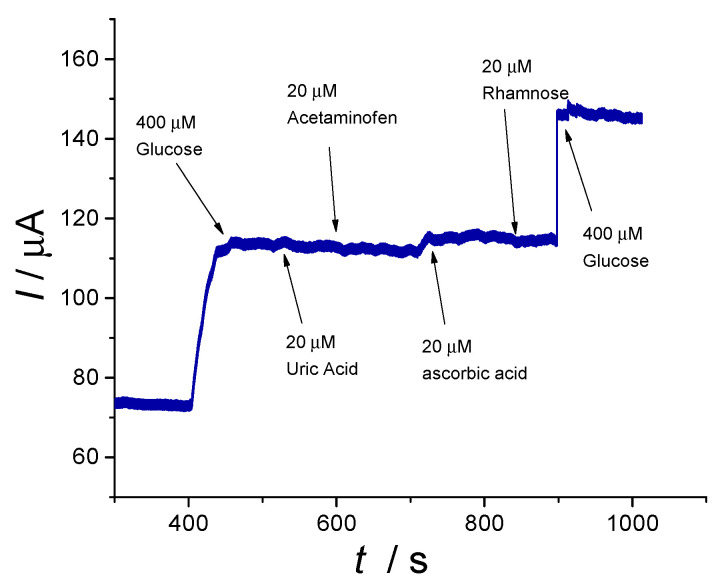
Amperometric responses in 0.1 M NaOH solution with standard additions every 100 s of 400 µM Glu, 20 µM UA, P, AA, R and 400 µM Glu (E_p_ = +0.55 V, 1100 s) for the Cu/Ni_f_ electrode (working electrode area of 25 mm^2^).

The current responses of Glu, UA, P, AA and R were also investigated at a constant potential of +0.55 V vs. Ag/AgCl, applying the same protocol as previously detailed, and the results are presented in Figure 10. 

After the signal stabilization, a standard addition of Glu was made corresponding to 400 µM Glu concentration. Different volumes of interfering agents were added every 100 s (final concentrations of 20 µM were achieved). Finally, after the interfering molecules, another Glu standard addition corresponding to 400 µM was performed. As expected from the CVs in the presence of Glu and P, respectively UA, the studied molecules did not have any influence on the electrochemical oxidation of the model molecule. A slight increase of the anodic current was observed when AA was injected, but with a minimum influence on the Glu signal, as can be seen from Figure 9. Overall, one can appreciate the use of the Cu/Ni_f_ electrode providing a promising response in the presence of the above-mentioned compounds, thus showing potential to be considered for real case scenarios.

### 3.4. Studies on Real Samples

**Figure 11:**
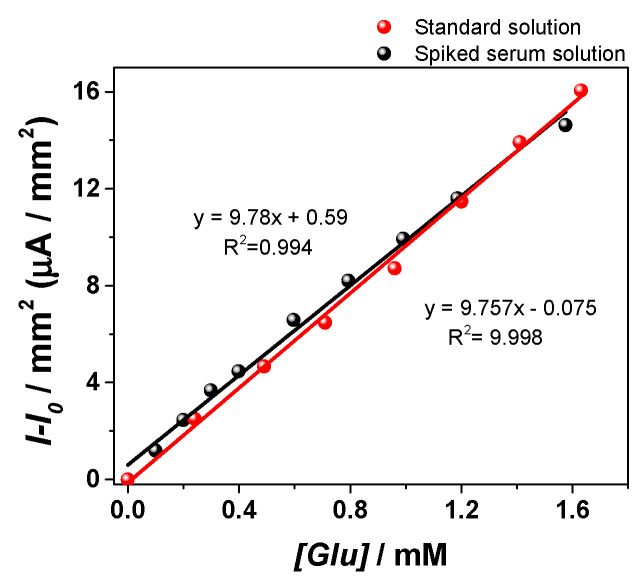
Calibration curve for the Cu/Nif on Cu electrode in the range of 0.24–1.63 mM Glu (red line) performed on standard solutions and on commercial human serum spiked with 0.1 M Glu (black line) (0.55 V, 0.1 s step potential, 1200 s analysis time).

The amperometric sensor was tested in commercial human serum spiked with 0.1 M Glu (Figure 11). The sample was diluted with 0.1 M NaOH (1:100 ratio) without filtration or any other pretreatment step. The I-I_0_/mm^2^ was plotted against Glu concentration and a proportional increase of the signal was observed. When the results were compared with those obtained on the standard solutions no significant differences were observed, suggesting that the sensor has the potential to be applied on real samples but only after a dilution of the real samples due to the fact that the linearity domain is 0.24–1.63 mM and it does not reach pathological hyperglycemia levels.

Table 4 presents other non-enzymatic Glu sensors involving different nanomaterials. Usually, they consist of various metallic morphologies sometimes associated with carbon-based nanomaterials.

The analytical performances of the developed sensors are similar with those obtained in the revised literature, and the linear range covers the physiological and pathological levels if the sample is priorly diluted with a compatible electrolyte.

The metallic porous electrodes, such as the one proposed in the present investigation, could be a low cost and adequate alternative to replace glucose oxidase without lacking in analytical properties. Likewise, the replacement of standard Glassy carbon electrodes with 3D Cu-based architectures could change the perspective of this approach and reduce the cost involved in the elaboration. The interference studies mentioned in our work are similar to those already reported and will have a positive input for further studies on this topic.

## 4. Conclusions

As a result of the performed investigations, 3D electroformed copper structures have been successfully synthesized, involving Ni foams as promotor of a nanoporous morphology. SEM images showed the growth of porous architectures usually consisting of pores having diameters in the range of 10 to 20 μm and an internal structure of the Cu deposit possessing a globular-like structure with an average diameter of about 100 nm.

The functionalization of the porous structure by applying Cu additional coatings facilitated an enhanced electroanalytical response related to the non-enzymatic detection of Glu using amperometry in the presence of common interfering species.

These results confirm that 3D Cu nanostructures may successfully act as electrode material for the sensing of Glu with promising potential for application in real case scenarios.

## Figures and Tables

**Table 1:** Electrolytes composition and operating conditions for electrochemical preparation of the 3D Cu nanoporous electrodes.

Coating System	Electrolyte Composition	Operating Conditions
Cu/Ni foam (denoted Cu/Ni_f_)	(i) 0.1 M NiCl_2_∙6H_2_O + 2M NH_4_Cl	pH = 4–4.5 T = 25 °C i = 1 A/cm^2^ time = 3 min.
	(ii) 0.8 M CuSO_4_∙5H_2_O + 0.6 M H_2_SO_4_	T = 25–30 °C i = 28–35 mA/cm^2^ time = 15–20 min.

**Table 2:** Fitting results of impedance spectra for Cu strip, Ni_f_ and Cu/Ni_f_ electrodes recorded in5 mM [Fe(CN)_6_]^3‒/4‒^ in 0.1 M NaOH solution using the equivalent circuits proposed in Figure 4.

Working Electrode Type	R_s_ / ohm cm^2^	R_ct_ / ohm cm^2^	C_dl_ /µF	R_W_ / ohm cm^2^	The Used Model
**Cu strip**	80.6	20	21.5	521	(A)
**Ni_f_**	111.2	4473	210.1	-	(B)
**Cu/Ni_f_**	185.4	10	291.5	348	(A)

**Table 3:** Double-layer capacitance, real surface area and corresponding surface roughness values for the investigated Cu electrodes calculated from the EIS data.

Working Electrode Type	CPE_dl_/µF	*A_real_*/cm^2^	σ
Cu strip	21.5	1.07	1.69
Ni_f_	210.1	10.5	16.67
Cu/Ni_f_	291.5	14.5	23.1

**Table 4:** Cu- and Ni-based electrochemical enzymeless sensors developed for Glu detection and their analytical parameters.

No.	Platform	Detection Method	LOD µM	Linearity µM	Interfering Species	Ref.
1.	NiNPs/ERGO	CA	0.04	0.5–244	Dopamine, UA, AA	[33]
2.	Cu-NW-CNT-BL/GCE	EIS, CV, CA	0.33 × 10^‒3^	10–2000	Sucrose, Fructose, Maltose, AA, Dopamine	[2]
3.	CuO nanorod	CA	1	1–1250	UA, AA Dopamine	[21]
4.	Cu-Cu_2_S nanocomposite/GCE	CV, CA	0.33	0.5–500	Fructose, Sucrose, AA, UA, Dopamine	[12]
5	CuO nanorods - AuNP	LSV	0.17	5–1325	AA, UA, Dopamine, Ureea, Sucrose	[13]
6.	Pt/Ni@rGO	CA	6.3	20–5000	AA, Phenol, Ethanol	[14]
7.	Cu/Ni Foam	CA	2	6–206 240–1630	UA, AA, R, P	This work
